# A tale of two tags: UFMylation counters ubiquitination for ciliary homeostasis

**DOI:** 10.1038/s41418-026-01676-y

**Published:** 2026-01-29

**Authors:** Jonathan N. Pruneda, Rune Busk Damgaard

**Affiliations:** 1 Department of Molecular Microbiology and Immunology, Oregon Health & Science University, Portland, OR USA; 2 Department of Biotechnology and Biomedicine, Technical University of Denmark, Kongens Lyngby, Denmark

**Keywords:** Cell biology, Biochemistry

Editorial on: *UFL1-mediated UFMylation antagonizes IFT88 ubiquitination and degradation to maintain ciliary homeostasis*

**Fig. 1:**
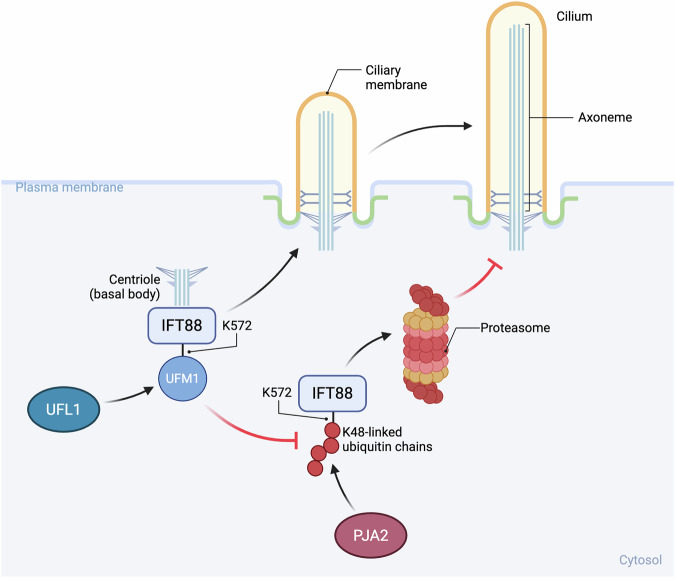
UFL1 is required for ciliary homeostasis. UFM1-specific ligase (UFL1) UFMylates IFT88 on K572. This UFMylation ensures ciliary integrity and function by preventing PJA2-mediated ubiquitination of the same residue and subsequent proteasomal degradation of IFT88. This mutually exclusive modification of K572 reveals an unexpected antagonism between UFMylation and ubiquitination in the regulation of ciliary homeostasis. Figure created in BioRender. Damgaard, RB. (2025) https://biorender.com/3x7ue19.

Posttranslational modification of proteins by ubiquitin and ubiquitin-like modifiers (UBLs) governs virtually all aspects of cell signalling and physiology, from proteostasis and cell-cycle control to responses to stress and infection [1]. While the molecular functions of ubiquitination are relatively well characterised, the roles of most UBL modifications remain elusive. In this issue of Cell Death & Differentiation, Wang et al. [2] uncover a striking example of crosstalk between these two systems. They show that ubiquitin fold modifier 1 (UFM1) [3] is conjugated to IFT88, a core component of the intraflagellar transport machinery required for cilia assembly, thereby shielding it from ubiquitination-dependent degradation (Fig. 1). This UFM1-mediated protection of IFT88 ensures ciliary integrity and tissue homeostasis.

Like the related ubiquitin system, UFM1 is covalently conjugated to protein substrates through a three-step enzymatic mechanism [3, 4]. The process is initiated by the E1 enzyme UBA5, which uses ATP to activate and transfer the UFM1 carboxy-terminus onto the catalytic cysteine of the E2 enzyme UFC1. The final step in the UFMylation cascade is transfer to the substrate protein facilitated by UFM1-specific ligase (UFL1) [3]. Genetic studies have linked defects in the UFMylation cascade to severe congenital neurological and developmental disorders, highlighting an important cellular role for this modification [3, 5]. Recent studies have implicated the endoplasmic reticulum (ER)-anchored protein UFBP1/DDRGK1 as an obligate heterodimeric partner of UFL1 [6–8]. Together, UFL1 and UFBP1/DDRGK1 support UFMylation of the ribosomal subunit RPL26 in response to ribosome stalling at the ER, which can then stimulate either ubiquitin-dependent disassembly through the ribosomal quality control pathway or clearance of the entire complex through ER-directed autophagy. Outside of ER-associated quality control pathways, UFMylation has also been tied to processes such as DNA repair, although it remains unclear if/how UFL1 and UFBP1/DDRGK1 facilitate these processes.

To further investigate the functions of UFMylation, Wang and colleagues [2] analysed an inducible UFL1-knockout mouse, thereby overcoming the embryonic lethality associated with constitutive UFL1 loss [9]. They noted reduced fertility in the mice, correlating with diminished sperm counts, shortened flagella, and impaired motility. Expanding their analysis, the authors identified widespread anatomical abnormalities in multiple ciliated tissues in the UFL1-deficient mice, including the reproductive, respiratory, neural, skeletal, renal, and ocular systems. Cilia are antenna- or hair-like organelles that project from the surface of cells. They can be non-motile mechano- and chemosensors involved in signal transduction, or they can be motile, beating in a coordinated manner to propel mucus or fluid along the cell surface [10, 11]. Using fluorescence and scanning electron microscopy, the authors demonstrated that motile cilia across these tissues were reduced both in length and density. These phenotypes were recapitulated in cultured cells, which exhibited shortened cilia and a reduced proportion of cells with non-motile cilia, indicating a general defect across cilium types. The authors thereby identified UFL1-mediated UFMylation as critical for maintaining ciliary integrity and function across tissues and cell types. Given the importance of cilia for tissue function and cell signalling, these findings raise intriguing questions about the contribution of ciliary dysfunction to the complex pathologies observed in patients with UFMylation defects. As mutations in the UFM1 pathway components cause skeletal and neurodevelopmental disorders, it is tempting to speculate that impaired ciliary function may, in part, underlie these conditions.

To unravel the molecular mechanisms by which UFL1 regulates ciliary homeostasis, the authors employed advanced microscopy techniques and identified that UFL1 localises to the centrosome, from where the cilium axoneme emerges. Proteomics analyses further revealed that UFL1 interacts with IFT88, a core component of the intraflagellar transport (IFT) complex that is required for ciliary maintenance. IFT88 was found to be UFMylated in a UFL1-dependent manner at lysine 572 (K572), and loss of this UFMylation led to accelerated IFT88 turnover. Mechanistically, the authors discovered that UFL1-dependent UFMylation of IFT88 antagonises its K48-linked polyubiquitination and subsequent proteasomal degradation mediated by the ubiquitin ligase PJA2. Thus, UFL1-mediated UFMylation and PJA2-mediated polyubiquitination compete for the same lysine (K572), establishing a regulatory switch that dictates IFT88 stability and ciliary homeostasis (Fig. 1). This mutually exclusive modification reveals an unexpected antagonism between UFMylation and ubiquitination, driving opposite outcomes for the substrate.

This newly uncovered UFM1-ubiquitin antagonism expands the repertoire of posttranslational crosstalk mechanisms that regulate protein stability and signalling. For instance, phosphorylation can promote ubiquitination by generating phosphodegrons recognised by SCF/βTrCP and other F-box ligases, thereby triggering phosphorylation-dependent ubiquitination and proteasomal degradation – an essential control mechanism across numerous signalling pathways [12]. Similarly, modification with the small ubiquitin-like modifier (SUMO) can promote ubiquitination via SUMO-targeted ubiquitin ligases (STUbLs) such as RNF4, which recognise SUMOylated substrates and append ubiquitin chains for substrate degradation [13]. In these cases, phosphorylation and SUMOylation serve as molecular cues that license ubiquitination and promote regulated protein degradation. By contrast, protein acetylation can antagonise ubiquitination by directly competing for the same lysine residues, thereby stabilising proteins through mutually exclusive modification [14]. In this context, the IFT88 K572 switch extends the paradigm of PTM- and UBL-dependent cross-regulation with ubiquitin to include UFMylation, positioning UFL1 as a molecular safeguard that shields a key ciliary component from ubiquitin-mediated destruction. Within ciliary biology, this form of cross-regulation is now linked to two substrates, with an earlier report from the same authors identifying a UFM1-ubiquitin switch controlling the kinesin family member KIF11 at the ciliary transition zone [15].

It will be interesting to uncover how this UFM1-ubiquitin switch is regulated, e.g., what cellular signals or stresses might flip the switch between protection and degradation? Identifying the pathways and sensors that control this modification cycle could reveal how cells dynamically tune ciliary organisation in response to physiological demands.
